# Whole-genome assembly of *Ganoderma leucocontextum* (Ganodermataceae, Fungi) discovered from the Tibetan Plateau of China

**DOI:** 10.1093/g3journal/jkab337

**Published:** 2021-09-29

**Authors:** Yuanchao Liu, Longhua Huang, Huiping Hu, Manjun Cai, Xiaowei Liang, Xiangmin Li, Zhi Zhang, Yizhen Xie, Chun Xiao, Shaodan Chen, Diling Chen, Tianqiao Yong, Honghui Pan, Xiong Gao, Qingping Wu

**Affiliations:** 1 School of Biology and Biological Engineering, South China University of Technology, Guangzhou 510006, China; 2 Guangdong Provincial Key Laboratory of Microbial Safety and Health, State Key Laboratory of Applied Microbiology Southern China, Institute of Microbiology, Guangdong Academy of Sciences, Guangzhou 510070, China; 3 Guangdong Yuewei Edible Mushroom Technology Co., Ltd., Guangzhou 510663, China

**Keywords:** *Ganoderma leucocontextum*, genome, Illumina, Nanopore, medicinal fungi, secondary metabolism, terpenoids

## Abstract

*Ganoderma leucocontextum*, a newly discovered species of Ganodermataceae in China, has diverse pharmacological activities. *Ganoderma leucocontextum* was widely cultivated in southwest China, but the systematic genetic study has been impeded by the lack of a reference genome. Herein, we present the first whole-genome assembly of *G. leucocontextum* based on the Illumina and Nanopore platform from high-quality DNA extracted from a monokaryon strain (DH-8). The generated genome was 50.05 Mb in size with an N50 scaffold size of 3.06 Mb, 78,206 coding sequences, and 13,390 putative genes. Genome completeness was assessed using the Benchmarking Universal Single-Copy Orthologs (BUSCO) tool, which identified 96.55% of the 280 Fungi BUSCO genes. Furthermore, differences in functional genes of secondary metabolites (terpenoids) were analyzed between *G. leucocontextum and Ganoderma lucidum*. *Ganoderma leucocontextum* has more genes related to terpenoids synthesis compared to *G. lucidum*, which may be one of the reasons why they exhibit different biological activities. This is the first genome assembly and annotation for *G. leucocontextum*, which would enrich the toolbox for biological and genetic studies in *G. leucocontextum*.

## Introduction

**Figure 1:**
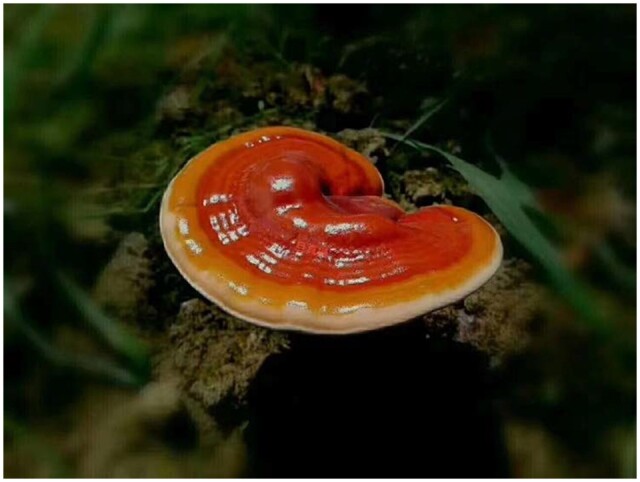
Fruiting bodies of *G. leucocontextum*. Scale bar: 1.0 cm.

*Ganoderma leucocontextum* (Figure 1) is a newly discovered prize medicinal species of *Ganoderma*, which was first found in Tibet, China, in 2015. The macroscopic morphological characteristics of the fruiting body of *G. leucocontextum* are highly similar to *Ganoderma lingzhi* (Li *et al.* 2015), which is widely cultivated and used in China (Cao *et al.* 2012), but there are major differences between these two species in terms of biological characteristics and pharmacological activity. For example, the hyphae of *G. leucocontextum* displayed an acid-tolerant characteristic; both the mycelium and the fruiting body grew at a lower temperature than that of *G. lingzhi* (HU *et al.* 2017; Mo Weipng *et al.* 2017).

Moreover, some novel bioactive compounds like triterpenes and ganoderols were isolated from *G. leucocontextum* in recent studies (Wang *et al.* 2015, 2017; Zhao *et al.* 2016a, 2016b; Chen *et al.* 2018a), which have been proved to have pharmacological activities such as anti-tumor (Li *et al.* 2019; Liu *et al.* 2018), antioxidant (PAN Jun *et al.* 2021), antihyperglycemic (Wang *et al.* 2015, 2017; Chen *et al.* 2019), hypolipidemic (Wang *et al.* 2017; Zhang *et al.* 2018b), neurodegenerative diseases prevention (Chen *et al.* 2018a; Xiong *et al.* 2016), anti-aging (Wang *et al.* 2019), and immune regulation (Gao *et al.* 2020). Therefore, it is classified as a high-quality category with higher price than the ordinary *Ganoderma* in Tibet, China (LIU *et al.* 2020; Shen *et al.* 2015). These have promoted the research of artificial cultivation of *G. leucocontextum* and realized the large-scale commercial cultivation since 2016. What is exciting is that it has been confirmed that the content of polysaccharide and triterpenoid of *G. leucocontextum* is significantly higher than that of *G. lingzhi* (HU *et al.* 2017).

Many microorganisms produce natural products like antimicrobials and drugs, while the mining of the genome can quickly screen and obtain new natural products (Gao *et al.* 2018; Blin *et al.* 2021). Genome research includes structural genomics, aiming to whole-genome sequencing, and functional genomics, aiming to explore gene function. The fungal genome initiative is the first well-known fungal genome project. It was initiated and launched by the mycology research group in conjunction with the Broad Institute of the United States in 2000, and then issued the white papers of the genome sequencing projects, which were specific to 15 and 44 species of fungi in 2002 and 2003, respectively (Li 2018). Up to May 2021, more than 2000 fungal genome projects, including 595 in basidiomycetes and 1240 in ascomycetes, have been completed and released on the JGI website (https://mycocosm.jgi.doe.gov/mycocosm/home). In terms of the well-known edible and medicinal fungi, such as *Ganoderma lucidum* (2012), *Flammulina velutipes* (2015), *Lentinula edodes* (2016), *Cordyceps guangdongensis* (2018), *Auricularia heimuer* (2019), *Hericium erinaceus* (2020), *Morchella sextelata* (2019), and *Agrocybe cylindracea* (2020), the Whole-Genome has been published and some functional genes were predicted and analyzed (Chen *et al.* 2012; Zeng *et al.* 2015; Shim *et al.* 2016; Zhang *et al.* 2018a; Mei *et al.* 2019; Yuan *et al.* 2019; Gong *et al.* 2020; Liang *et al.* 2020). Despite progresses have been made toward understanding the cultivation and efficacy, the genome-wide association studies of *G. leucocontextum* has not been systematically performed. The *G. leucocontextum* strain in this study was from Nyingchi, Tibet, which was rich in triterpenes compared to *G. lucidum* (HU *et al.* 2017). In order to clarify genetic and physiological background of *G. leucocontextum*, whole-genome sequencing was carried out by Oxford Nanopore technologies; furthermore, functional annotation and gene clusters of secondary metabolites were predicted based on public databases.

## Methods and materials

### Fungal strains and nucleic acid extraction

In this study, the dikaryon strain (HMGIM-I160015) was isolated from the fruiting body of *G. leucocontextum* that was collected in Nyingchi by HU Huiping. The monokaryon strain DH-8 was isolated from HMGIM-I160015 using the protoplast-derived method (Li *et al.* 2021) and preserved in Institute of Microbiology, Guangdong Academy of Sciences. Vegetative mycelium of DH-8 was cultured on Potato Dextrose Agar (PDA) medium (20% potato, 2% glucose, 2% agar, 0.3% KH_2_PO_4_, 0.15% MgSO_4_, trace of vitamin B_1_) with cellophane at 25°C in darkness for 7 days. Then, the mycelia were frozen in liquid nitrogen and ground to powder for genomic DNA extraction. Genomic DNA was extracted by QIAGEN^®^ Genomic DNA extraction kit (Cat#13323, QIAGEN) according to the manufacturer’s instructions. The extracted DNA was detected by NanoDrop™ One UV-Vis spectrophotometer (Thermo Fisher Scientific, USA) for DNA purity (OD_260/280_ ranging from 1.8 to 2.0 and OD_260/230_ is between 2.0 and 2.2) and then Qubit^®^ 3.0 Fluorometer (Invitrogen, USA) was used to quantify DNA accuracy. Total RNA was extracted from the fruiting body tissue by using Plant RNA Purification Reagent, the concentration and purity of the extracted RNA were detected using Nanodrop2000, and the RNA integrity number (RIN) value was determined by Agilent2100.

### *De novo* sequencing and assembly

*De novo* genome sequencing of DH-8 was performed with a 20-k and 350-bp library size using the Nanopore and Illumina sequel platform at Biomarker Technologies Corporation (Beijing, China), respectively (Wick *et al.* 2019). The filtered subreads were assembled using NECAT software (https://github.com/xiaochuanle/NECAT). And then the assembled genome was corrected in contrast to the data of the Illumina using Pilon v1.22 (Walker *et al.* 2014) software, resulting in a more accurate final genome. Burrows-Wheeler Aligner (bwa) (Li and Durbin 2009) and Benchmarking Universal Single-Copy Orthologs (BUSCO) v3.0.1 (Simão *et al.* 2015) were used to assess the completeness of genome assembly. RNA sequencing was performed using Illumina HiSeq xten/NovaSeq 6000 sequencer (2 × 150 bp read length), and standard bioinformatics analyses at Shanghai Major Biomedical Technology Co., Ltd. (Shanghai, China). The genome data of DH-8 have been submitted to NCBI, accession number was AHKGY000000000.

### Genomic component analysis

#### Repeat sequence prediction

LTR_FINDER v1.05 (Xu and Wang 2007), MITE-Hunter (Han and Wessler 2010), RepeatScout v1.0.5 (Price *et al.* 2005), and PILER-DF v2.4 (Edgar and Myers 2005) were applied to construct the repetitive sequence database of the genome of *G. leucocontextum* based on the structure and *ab initio* prediction, then the predicted database was categorized by PASTEClassifier (Wicker *et al.* 2007), and merged as the final repeated sequence database with Repbase (Jurka *et al.* 2005). The repetitive sequences of *G. leucocontextum* were predicted by RepeatMasker v4.0.6 (Chen 2004) based on the constructed repeated sequence database.

#### Protein-coding genes prediction

Gene prediction was conducted through a combination of *ab initio* prediction, homology-based prediction, and transcriptome-based prediction methods. In detail, *ab initio* gene predictions were performed using Genscan (Burge and Karlin 1997), Augustus v2.4 (Stanke and Waack 2003), GlimmerHMM v3.0.4 (Majoros *et al.* 2004), GeneID v1.4 (Blanco *et al.* 2007), and SNAP (version 2006-07-28) (Korf 2004). Coding gene structures were predicted by GeMoMa v1.3.1 (Keilwagen *et al.* 2016) based on an alignment of orthologous proteins. Data analysis of RNA-seq Raw reads from Illumina sequencing was trimmed and assessed for quality with Fastp (Chen *et al.* 2018b), the qualified data were aligned to the genome by Hisat2 (Siren *et al.* 2014), the aligned reads were assembled into transcripts using Stringtie (Kovaka *et al.* 2019), and then open reading frames were predicted using PASA (Program to Assemble Spliced Alignments) (Haas *et al.* 2003). Finally, EVidenceModeler (EVM) (Haas *et al.* 2008) was used to produce an integrated gene set, the transposable elements (TEs) were removed using TransposonPSI (Urasaki *et al.* 2017) package (http://transposonpsi.sourceforge.net/), and the miscoded genes were further filtered. All the above software were used with the default parameters.

#### NcRNAs and pseudogene annotation

To obtain the non-coding RNA (ncRNA) of the genome, tRNAscan-SE (Lowe and Eddy 1997) was used to predict transfer RNAs (tRNAs) with eukaryote parameters. Infernal 1.1 (Nawrocki and Eddy 2013) and RNAmmer (Lagesen *et al.* 2007) were used to predict ribosomal RNAs (rRNAs) and other RNAs based on Rfam (Griffiths-Jones *et al.* 2005) databases. Pseudogenes were defined as any gene that had a loss of function mutation anywhere within the coding sequence, though they have similar sequences to functional genes and have lost their original functions due to mutations such as insertions and deletions; however, there are still potential functions of affecting protein (Winglee *et al.* 2016). The predicted protein sequences were aligned to the protein sequence included in the Swiss-Prot database by using the GenBlastA (She *et al.* 2009) to find homologous gene sequences (possible genes) in the genome. Then, the immature termination codon and frameshift mutation in the gene sequences were searched to obtain pseudogenes by using the software GeneWise (Birney *et al.* 2004).

### Genome functional annotation

The predicted genes were aligned against the functional databases such as Eukaryotic Orthologous Groups (KOG) (Galperin *et al.* 2015), Kyoto Encyclopedia of Genes and Genomes (KEGG) (Kanehisa and Goto 2000), Swiss-Prot (Stanke and Waack) (Stanke *et al.* 2006), TrEMBL (Boeckmann *et al.* 2003), Non-Redundant Protein Sequence Database (NR) (Deng *et al.* 2006), transporter classification database (TCDB) (Saier *et al.* 2006), and the pathogen–host interaction factor database (PHI) (Winnenburg *et al.* 2006) by BLAST (Altschul *et al.* 1997) to obtain the results of gene functional annotation. Based on the blast results of NR database, the software Blast2Go (Conesa *et al.* 2005) was applied to annotate the function of Gene Ontology (GO) (Ashburner *et al.* 2000) database. In addition, gene function annotation analysis was performed on Clusters of Orthologous Groups (COG), KEGG metabolic pathway enrichment analysis, and GO function enrichment analysis. Hmmer (Eddy 1998) was used for functional annotation of carbohydrate-related enzymes based on the database of carbohydrate-active enzymes (CAZymes) (Cantarel *et al.* 2009). Additionally, the online software *antiSMASH* (https://antismash.secondarymetabolites.org/#!/start) (Medema *et al.* 2011) was employed to predict gene clusters of secondary metabolites. KEGG Mapper (Reconstruct Pathway) (Minoru Kanehisa and Sato 2019) was also used to comparative analysis of pathway in metabolism of terpenoids and polyketides between *G. leucocontextum and G. lucidum*.

## Results and discussion

### Genome assembly and evaluation

A total of 47.5 μg DNA were obtained, in which A_260/280_ was 1.88 and OD_260/230_ was 2.26 according to Nanodrop detection, indicating that the extracted DNA was pollution-free and there was no protein or other contamination. A total of 8.58 Gb and 15.45 Gb raw data were obtained using the Illumina and Nanopore’s sequel platform, respectively. A total of 14.42 Gb clean data from Nanopore were obtained by filtering ploy-N, adapters, and low-quality reads from raw data. The quality statistics on the raw data and clean data was shown in Supplementary Table S1. The assembled genome was 50.05 Mb with a GC content of 55.85%; the sequencing depth was 288.16× and consisted of 58 scaffolds with an N50 of 3.06 Mb (Supplementary Table S2). Clean data from Illumina were aligned to the assembled genome by bwa (Li and Durbin 2009) to assess the genome assembly quality, and the coverage of assembled genome was 96.13%. The result of assessment for genome integrity by BUSCO (Simão *et al.* 2015) analyses was 96.55% (Table 1), indicating that vast majority of the conserved core genes of fungi were predicted, which revealed the high reliability of the prediction. Compared with similar species of the gene of *Ganoderma*, the genome size of *G. leucocontextum* was larger than *G. lucidum* (43.3 Mb), *G. tsugae* (45.5 Mb), and *G. sinense* (48.96 Mb) (Table 2). The number of scaffolds and Contig N50 revealed that we got the better genome assembly of *G. leucocontextum* in this study.

**Table 1:** The quality assessment for genome assembly of *G. leucocontextum*

Quality assessment	Values
Library*^a^*	350 bp
Mapped (%)*^b^*	99.39
Properly mapped (%)*^c^*	97.66
Coverage (%)*^d^*	96.13
Depth (X)*^e^*	74.36
Complete BUSCOs (C)*^f^*	280 (96.55%)
Complete and single-copy BUSCOs (S)*^g^*	276 (95.17%)
Complete and duplicated BUSCOs (D)*^h^*	4 (1.38%)
Fragmented BUSCOs (F)*^i^*	1 (0.34%)
Missing BUSCOs (M)*^j^*	9 (3.10%)
Total lineage BUSCOs*^k^*	290

a Represents Illumina sequencing library size.

b Represents the percentage of clean reads mapped to the genome assembly of *G. leucocontextum* to all clean reads.

c Represents the paired-end sequencing; sequences were all located on the genome assembly of *G. leucocontextum* and the distance was consistent with the length distribution of the sequenced fragments.

d Represents genome coverage of data from Illumina sequence.

e Represents genome coverage depth of data from Illumina sequence.

f Represents the number and percentage of complete genes found in the database (contains 290 conserved core genes of fungi).

g Represents the number and percentage of complete single-copy genes.

h Represents the number and percentage of complete duplicated genes.

i Represents the number of predictions for incomplete genes.

j Represents the unpredicted number of genes.

k Represents the number of conserved gene sets in fungi from the database of fungi_odb9.

**Table 2:** Genomic comparison of important species of *Ganoderma*

*Ganoderma sp.*	Strain	GenBank assembly accession	Genome size (Mb)	Number of scaffolds	GC%	Genome coverage	Assembly level	Contig N50/bp	Sequencing technology
*G. leucocontextum*	HMGIM-I160015	AHKGY000000000	50.05	58	55.85	288.16x	Scaffold	3,064,430	Illumina, Nanopore
*G. lucidum*	BCRC 36111	GCA_012655175.1	48.91	173	55.1	97.73x	Contig	1,281,108	PacBio
*G. lucidum*	BCRC 37177	GCA_000338035.1	44.08	3,275	55.5	824x	Contig	63,041	Illumina
*G. lucidum*	G.260125-1	GCA_000271565.1	43.29	82	56.1	440x	Scaffold	649,708	Illumina
*G. lucidum*	Xiangnong No.1	GCA_000262775.1	39.95	634	55.3	70x	Scaffold	80,796	Illumina
*G. tsugae*	s90	GCA_003057275.1	45.50	6,638	—	101.0x	Scaffold	11,659	Illumina HiSeq
*G. sinense*	ZZ0214-1	GCA_002760635.1	48.96	69	55.6	500.0x	Scaffold	753,893	454; Illumina HiSeq
*G. multipileum*	BCRC 37180	GCA_000338015.1	46.38	6,173	55.3	824x	Contig	50,471	Roche/454; Illumina/ABI
*G. sp.*	BRIUMSc	GCA_008694245.1	52.28	12,158	55.6	99.35x	Contig	6,197	Illumina
*G. boninense*	NJ3	GCA_001855635.1	60.33	18,903	55.9	20.0x	Contig	6,116	Illumina HiSeq; 454
*G. boninense*	G3	GCA_002900995.2	79.19	495	55.9	50.0x	Contig	272,644	PacBio

Data of *G. leucocontextum* were from this study; other data were from NCBI.

### Genome structure analysis

#### Repeat sequence annotation

The total number of repeat sequences was 4231, covering 12.64% of the genome. In detail, TEs of DNA and RNA account for 1.02% and 8.11%, respectively. Whereas the proportion of long terminal repeats was 5.06%, the proportion of unknown repeat sequences was 3.26%.

#### Coding protein genes prediction

The annotations of 13,390 protein-coding genes were supported by the public databases. The total sequences length for all the protein-coding genes were 29,573,839 bp, average of gene length was 2,208.65 bp, number of exon and intron were 82,221 and 68,831, respectively. Detailed gene information statistics were shown in Table 3. The number of predicted genes by homology and transcriptome prediction was 12,837, accounting for 95.87% of the annotated genes, which reveals high quality of the gene prediction. The annotations with NR, COG, KEGG, *etc.* were shown in Supplementary Table S3.

**Table 3:** Gene information statistics of *G. leucocontextum*

Gene statistics	Values
Gene number	13,390
CDs number	78,206
Exon number	82,221
Intron number	68,831
Gene length	29,573,839
CDs length	19,331,970
Exon length	23,157,769
Intron length	6,416,070
Average gene length	2,208.65
Average CDs length	247.19
Average exon length	281.65
Average intron length	93.21
Average CDs number	5.84
Average exon number	6.14
Average intron number	5.14

#### NcRNAs and pseudogene annotation

NcRNAs have no or limited protein-coding capacity but as potent and multifunctional regulators (Lekka and Hall 2018), including tRNAs, rRNAs, and long ncRNAs (lncRNAs). According to the structural characteristics of ncRNAs, different strategies are used. For ncRNA, 350 tRNAs, 78 rRNAs, and 72 other ncRNAs were predicted. The number of tRNA family based on the different anticodon was 51. Among the tRNAs, 15 were pseudo anticodons, 1 was undetermined anticodon, and the remaining anticodon tRNAs correspond to the 20 common amino acid codons. A total of 180 pseudogenes were predicted, the total size of Pseudogenes was 443,628 bp, and the average length was 2,464.6 bp.

### Genome functional annotation

A total of 12,724 non-redundant genes were annotated from the public databases. Among the annotated genes, 12,703 genes were annotated in the NR database, followed by 12,566 (TrEMBL), 7,894 (Pfam), 6,345 (Swiss-Prot), 5,658 (KOG), 3,401 (KEEG), and 3,110 (GO) (Table 4). These homologous protein genes represent 95.03% of the predicted genes of assembled genome.

**Table 4:** Functional annotation of *G. leucocontextum* genes from public databases

Public database	Number of genes	Percentage
GO	3,110	24.4
KEGG	3,401	26.7
KOG	5,658	44.5
Pfam	7,894	62.0
Swiss-Prot	6,345	49.9
TrEMBL	12,566	98.8
Nr	12,703	99.8
All annotated	12,724	100.0

#### Genomics analysis of KOG annotations

**Figure 2:**
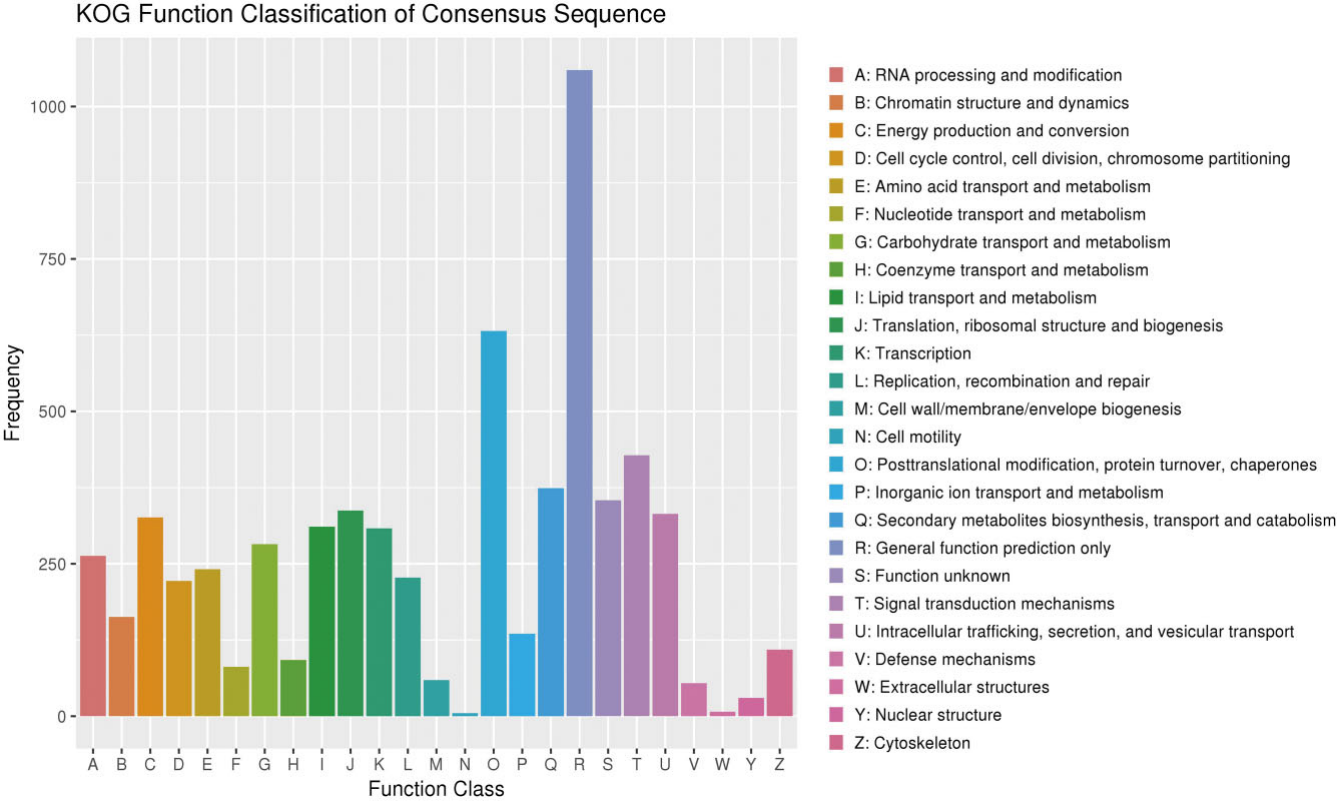
Annotated result classification chart by KOG database.

KOG is a gene orthology database for eukaryotes. In this study, 5658 genes were assigned to the KOG categories while the majority of genes was classified into the “General function prediction only,” followed by “Posttranslational modification, protein turnover, chaperones,” “Signal transduction mechanisms,” “Secondary metabolites biosynthesis, transport, and catabolism.” There were fewer genes in “Nuclear structure” and “Defense mechanisms,” and much fewer genes in “Cell motility” and “Extracellular structures” (Figure 2). A total of 1842 (32.55% of the total) predicted genes were involved in metabolic processes, and 374 (6.61% of the total) predicted genes were related to “secondary metabolites biosynthesis, transport, and catabolism.” The closely related species *G. lucidum* has the similar number of functional genes by KOG annotation (Chen *et al.* 2012). In addition, the number of genes related to “Secondary metabolites biosynthesis, transport, and catabolism” in *G. leucocontextum* was much more than that of mycorrhizal and straw-rotting fungus, such as *Laccaria bicolor* (Martin *et al.* 2008) and *Agaricus bisporus* (Kerrigan *et al.* 2013), and also it was much more than that of *G. lucidum* (Liu *et al.* 2012). Although the number of functional genes cannot determine the number of active ingredients, the result reveals the possible genetic basis of *G. leucocontextum* being rich in secondary metabolites. According to the results of previous research (Xia *et al.* 2014; Kladar *et al.* 2016), the species which belongs to the genus of *Ganoderma* was rich in triterpenes and other metabolites, and this seemed to be consistent with the results of the KOG annotation.

#### Genomics analysis of GO annotations

**Figure 3:**
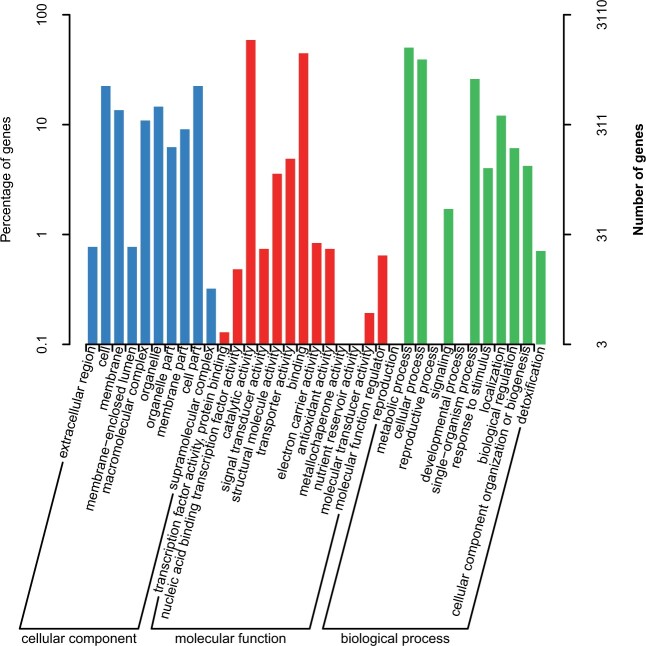
Annotated result classification chart by GO database.

To shed light on the potential roles of the predicted genes, GO enrichment analysis was performed. A total of 3110 GO annotations were matched and distributed in three functional categories: “biological process,” “cellular components,” and “molecular function.” The top four categories of GO were “catalytic activity,” “metabolic process,” “binding,” and “cellular process” (Figure 3), similar to *Hericium erinaceus* (Gong *et al.* 2020) and *Auricularia heimuer* (Yuan *et al.* 2019) but different to *Agaricus bisporus* (Kerrigan *et al.* 2013). On the other hand, the number of genes associated with the categories of “Nutrient reservoir activity,” “Reproduction,” “Reproductive process,” and “developmental process” was fewer, which may reflect from one side why *G. leucocontextum* can only distributes in a narrow area.

#### Genomics analysis of KEEG annotations

**Figure 4:**
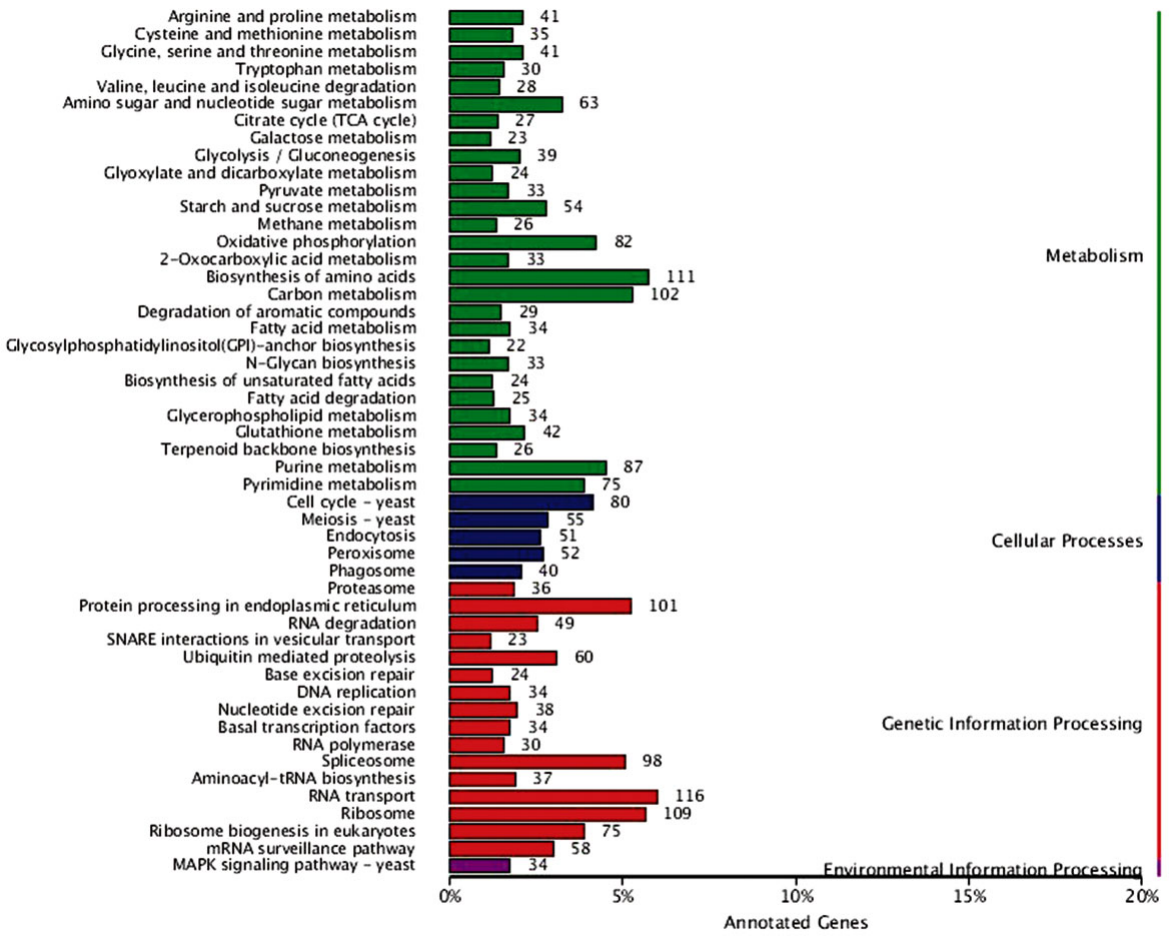
Annotated result classification chart by KEEG database.

KEGG is a comprehensive database that collects information on genomes, pathways, and compounds of organisms, which can help to further understand the gene functions in *G. leucocontextum*. According to the result of KEGG function annotation, 3401 genes were annotated to four physiological processes including “Metabolism,” “Cellular processes,” “Genetic information processing,” and “Environmental information processing” (Figure 4). In the second layer of KEGG pathway terms, we found that *G. leucocontextum* had much more genes associated to “RNA transport” (116 genes), “Biosynthesis of amino acids” (111 genes), “Ribosome” (109 genes), “Carbon metabolism” (102 genes), and “Protein processing in endoplasmic reticulum” (101 genes). These results may reveal the genetic basis of *G. leucocontextum* being rich in the secondary metabolites.

### Carbohydrate-active enzymes annotation

**Figure 5:**
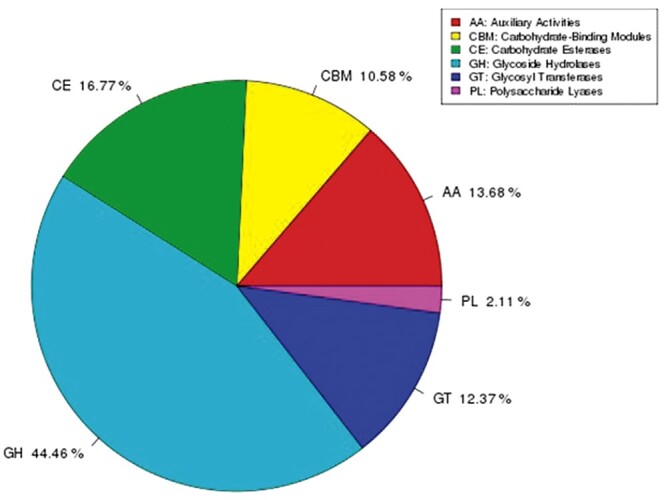
The CAZymes annotation of *G. leucocontextum*.

The CAZymes database includes glycoside hydrolases (GHs), glycosyltransferases (GTs), polysaccharide lyases (PLs), carbohydrate esterases (CEs), and auxiliary activities (AAs). Genes were annotated against the CAZy database to further understand the carbohydrate degradation capacity of *G. leucocontextum*. A total of 614 genes were assigned to CAZymes families as defined in CAZy database (Table 5 and Figure 5). Like other species’ CAZymes, the GHs were the most abundant enzymes of *G. leucocontextum*, in which 273 genes were predicted (Table 6). Furthermore, *G. leucocontextum* had more CAZymes genes than other fungi, including wood-rotting fungi [*G. lucidum* (Liu *et al.* 2012), *Auricularia heimuer* (Yuan *et al.* 2019), and *H. erinaceus* (Gong *et al.* 2020)], straw-rotting fungi (*M. sextelata*) (Mei *et al.* 2019), Mycorrhizal fungi (*L. bicolor*), and entomogenous fungi (*Cordyceps militaris*) (Zheng *et al.* 2011). It was interesting to note that 42 genes in *G. leucocontextum* were annotated to GH16, which was associated with the growth and development of fungi, and plays an important role in drought and other stresses (Sun *et al.* 2019), while 30 genes were assigned to GH18, which mainly contains the function of catalyzing the decomposition of chitin, but the number was less than *G. lucidum*, which has the highest genes number (40) annotated to GH18 among the known basidiomycetes (Liu *et al.* 2012). In particular, a number of CEs of *G. leucocontextum* were more than other fungus; 50 genes were annotated to CE10, which is related to the activities in aryl esterase, carboxyl esterase, acetylcholinesterase, cholinesterase, sterol esterase, and brefeldin A esterase, this also maybe the genetic basis of *G. leucocontextum* for the abundant sterols and other secondary metabolites.

**Table 5:** Annotation results of carbohydrate-active enzymes

Type	Number	Percentage
AAs	84	13.68
CBMs	65	10.58
CEs	103	16.77
GHs	273	44.46
GTs	76	12.37
PLs	13	2.11

**Table 6:** The gene distribution of CAZymes in six different fungi

Nutritional ecological type	Species	The proportion of match number and percentage						
		GHs	GTs	PLs	CEs	CBMs	AAs	Totals
Wood-rotting fungi	*G. leucocontextum*	273 (44.46%)	76 (12.37%)	13 (2.11%)	103 (16.77%)	65 (10.58%)	84 (13.68%)	614
Wood-rotting fungi	*G. lucidum*	288 (58.90)	70 (14.31%)	10 (2.04%)	30 (6.13)	53 (10.84)	38 (7.77%)	489
Wood-rotting fungi	*A. heimuer*	106 (31.55%)	29 (8.63%)	14 (4.17%)	22 (6.55%)	103 (30.65%)	66 (18.45%)	340
Wood-rotting fungi	*H. erinaceus*	161 (47.21%)	59 (17.30%)	7 (2.05%)	26 (7.62%)	4 (1.17%)	84 (24.63%)	341
Straw-rotting fungi	*M. sextelata*	159 (47.60%)	41 (12.28%)	20 (5.99%)	13 (3.89%)	57 (17.07%)	44 (13.17%)	334
Mycorrhizal fungi	*L. bicolor*	112 (51.85%)	41 (18.98)	5 (2.31)	5 (2.31%)	21 (9.72%)	32 (14.81%)	216
Entomogenous fungi	*C. militaris*	159 (50.15%)	84 (26.49%)	4 (1.26%)	13 (4.10%)	2 (0.63%)	55 (17.35%)	317

### Secondary metabolism analysis

**Figure 6:**
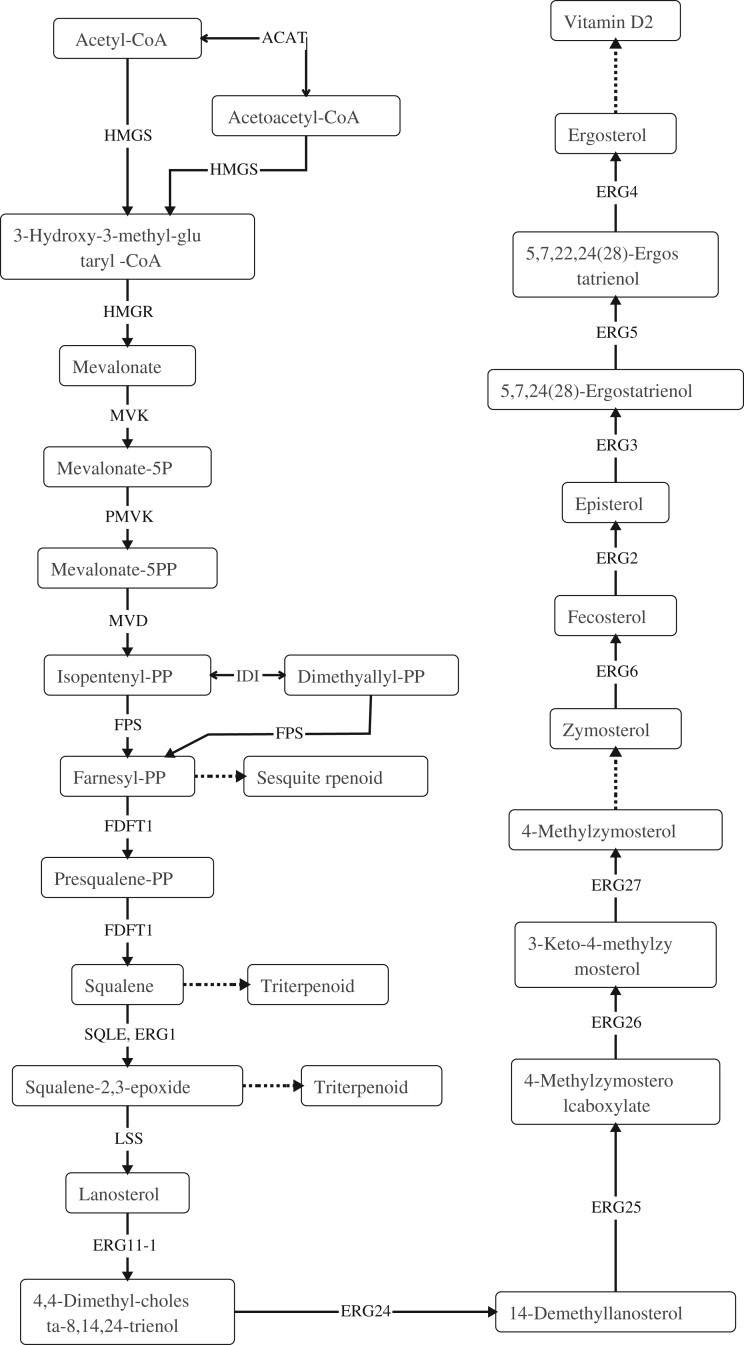
Triterpenoid and ergosterol biosynthesis pathways of *G. leucocontextum*.

**Figure 7:**
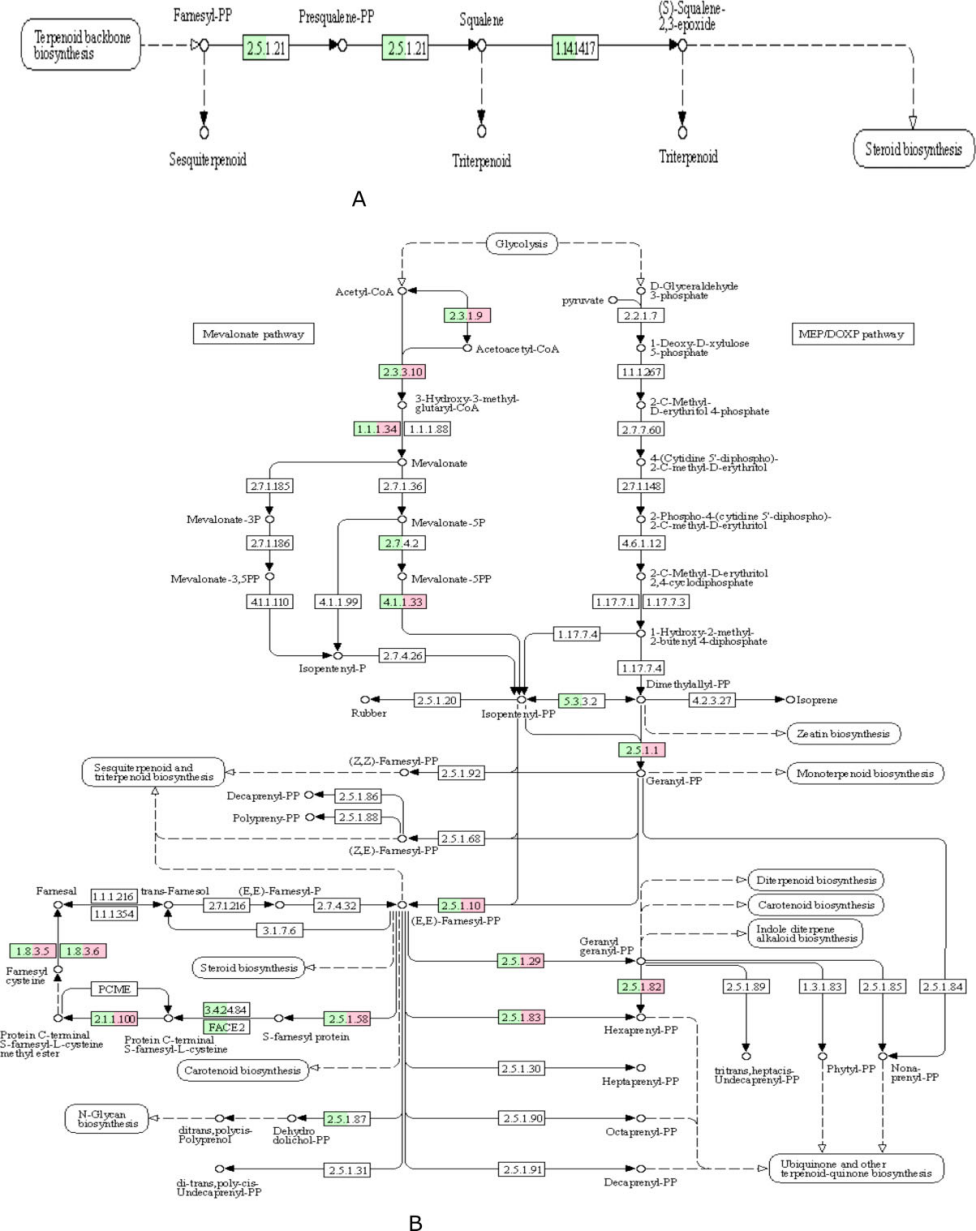
Enzyme in *G. leucocontextum and G. lucidum* involved in the pathway of Sesquiterpenoid and triterpenoid biosynthesis (map00909, A) and Terpenoid backbone biosynthesis (map00900, B). The colored box indicated existing homologous genes of the enzyme; green represent *G. leucocontextum*, red represent *G. lucidum*, white box means not, the same below.

Mushroom have been widely used as food and medicine in different part of the world for centuries. The main reason was that mushroom can not only be used as the nutritional source, such as dietary fiber, proteins, fats, amino acids, minerals, and vitamins, but also be used as potential pharmaceutical applications owing to the bioactive metabolites, including polysaccharides, terpenoids, fungal immunomodulatory proteins, and many other low-molecular-weight substances, which were widespread in the fruiting body (Elsayed *et al.* 2014; Zhao *et al.* 2020). The encoding genes for the biosynthesis of these active compounds were often organized as biosynthetic gene clusters (Blin *et al.* 2019). Previous studies on functional gene clusters, such as terpene synthases (Chen *et al.* 2011; Quin *et al.* 2014), non-ribosomal peptide synthetases (NRPS) (Finking and Marahiel 2004) (Schwarzer *et al.* 2003), and polyketide synthases (PKS) (Sims and Schmidt 2008; Lackner *et al.* 2012), have provided the references of gene clusters for genome mining. By using antiSMASH, one NRPS, one beta-lactone, three T1PKS, six NRPS-like, and ten terpene gene clusters were identified in the genome of *G. leucocontextum*; the details of secondary metabolite gene clusters are listed in Supplementary Table S4. This revealed that abundant terpenoid gene synthesis clusters may be the genetic basis for fruiting bodies of *G. leucocontextum* to produce rich terpenoids. Triterpenoids are a highly diverse group of natural products that are widely distributed in eukaryotes, and many triterpenoids have beneficial properties for human health. *Ganoderma lucidum* has the most diverse and abundant triterpenoid content of all examined fungi (Bishop *et al.* 2015). Ergosterol compounds are one of the major groups of therapeutic compounds in *Ganoderma* species. Previous studies in *G. lucidum* have identified 24 key genes involved in the biosynthesis of ergosterol compounds (Chen *et al.* 2012). In this study, Terpenoid backbone biosynthesis (26 genes) and Sesquiterpenoid and triterpenoid biosynthesis (3 genes) were identified in the genome of *G. leucocontextum*. Compared to *G. lucidum* (Chen *et al.* 2012), *G. leucocontextum* almost have all the genes required in the whole synthesis pathway of ganoderic acids and ergosterol, which were the two important secondary metabolites except for the genes ERG11-2, but it can be substituted by ERG11-1 (Table 7). Based on previous research (Cai *et al.* 2021), the triterpenoid and ergosterol biosynthesis pathways of *G. leucocontextum* were deduced (Figure 6). Considering that terpenoids are bioactive natural products widespread in fungi, especially in the fruiting body of *G. lucidum* (Su *et al.* 2020), we compared and analyzed the genes and enzymes involved in the pathways of metabolism of terpenoids and polyketides between *G. leucocontextum and G. lucidum* (Table 8). The core genes for C10–C20 isoprenoid biosynthesis (non-plant eukaryotes) were all present in the two species, and the pathway of “Sesquiterpenoid and triterpenoid biosynthesis” was intact in *G. leucocontextum* but incomplete in *G. lucidum* (Figure 7). The results were not quite similar to previous research results (Gu *et al.* 2017), that is, genes of isopentenyl diphosphate isomerase (IDI) and mevalonate kinase were present in *G. lucidum*. We suspect that there may be two reasons: first, poor-quality assembly of the *G. lucidum* genome hamper downstream analyses; second, there may be other metabolic pathways being used for the synthesis of terpenoids and polyketides in *G. lucidum*. Irrespectively, we have demonstrated that there is a difference in the active ingredients between the two species as they show different effects in cell experiments (the results have not published).

**Table 7:** Genes of ganoderic acids and ergosterol biosynthesis in *G. leucocontextum* and *G. lucidum*

Secondary metabolite	Gene name	Gene number	
		*G. lucidum*	*G. leucocontextum*
Ganoderic acids	AACT-1	GL23502	EVM0010239.1
	AACT-2	GL26574	EVM0000725.1
	FPS-1	GL22068	EVM0004420.2
	FPS-2	GL25499	EVM0009008.1
	HMGR	GL24088	EVM0008852.1
	HMGS	GL24922	EVM0003163.1
	IDI	GL29704	EVM0010983.1
	LSS	GL18675	EVM0005702.1
	MVD	GL25304	EVM0004601.1
	MVK	GL17879	EVM0008302.1
	PMVK	GL17808	EVM0009209.1
	SE	GL23376	EVM0008824.1
	SQS	GL21690	EVM0010547.1
Ergosterol	ERG11-1	GL26139	EVM0001186.1
	ERG11-2	GL22375	/
	ERG2	GL22516	EVM0010304.1
	ERG24	GL23832	EVM0011570.1
	ERG25	GL23074	EVM0000338.1
	ERG26	GL16838	EVM0012405.1
	ERG27	GL22371	EVM0011171.1
	ERG3	GL26052	EVM0000544.3
	ERG4	GL21870	EVM0000917.1
	ERG5	GL30444	EVM0010271.1
	ERG6	GL18323	EVM0002811.1

**Table 8:** Pathway of metabolism of terpenoids and polyketides in *G. leucocontextum and G. lucidum*

Pathway of metabolism of terpenoids and polyketides	Gene name	Definition	KO term	EC number	*G. leucocontextum*	*G. lucidum*
Terpenoid backbone biosynthesis/map00900	ACAT, atoB	Acetyl-CoA C-acetyltransferase	K00626	EC:2.3.1.9	Present	Present
	E2.3.3.10	Hydroxymethylglutaryl-CoA synthase	K01641	EC:2.3.3.10	Present	Present
	HMGCR	Hydroxymethylglutaryl-CoA reductase (NADPH)	K00021	EC:1.1.1.34	Present	Present
	E2.7.4.2, mvaK2	Phosphomevalonate kinase	K00938	EC:2.7.4.2	Present	Absent
	MVD, mvaD	Diphosphomevalonate decarboxylase	K01597	EC:4.1.1.33	Present	Present
	idi, IDI	Isopentenyl-diphosphate Delta-isomerase	K01823	EC:5.3.3.2	Present	Absent
	FDPS	Farnesyl-diphosphate synthase	K00787	EC:2.5.1.1, EC:2.5.1.10	Present	Present
	PCYOX1, FCLY	Prenylcysteine oxidase/farnesylcysteine lyase	K05906	EC:1.8.3.5, EC:1.8.3.6	Present	Present
	ICMT, STE14	Protein-*S*-isoprenylcysteine *O*-methyltransferase	K00587	EC:2.1.1.100	Present	Present
	STE24	STE24 endopeptidase	K06013	EC:3.4.24.84	Present	Absent
	RCE1, FACE2	Prenyl protein peptidase	K08658	EC:3.4.22.-	Present	Absent
	FNTB	Protein farnesyltransferase subunit beta	K05954	EC:2.5.1.58	Present	Present
	DHDDS, RER2, SRT1	Ditrans, polycis-polyprenyl diphosphate synthase	K11778	EC:2.5.1.87	Present	Absent
	GGPS1	Geranylgeranyl diphosphate synthase, type III	K00804	EC:2.5.1.29	Present	Present
	hexPS, COQ1	Hexaprenyl-diphosphate synthase	K05355	EC:2.5.1.82, EC:2.5.1.83	Present	Present
Sesquiterpenoid and triterpenoid biosynthesis/map00909	FDFT1	Farnesyl-diphosphate farnesyltransferase	K00801	EC:2.5.1.21	Present	Absent
	SQLE, ERG1	Squalene monooxygenase	K00511	EC:1.14.14.17	Present	Absent

### Conclusion

As a newly discovered prize medicinal mushroom, the pharmacological activity and cultivation characteristics of *G. leucocontextum* have been studied. However, the study of functional and biological properties at the genome level remains unknown. The genome of *G. leucocontextum* in this study provided the gene information and laid the foundation for further understanding of the reasons behind its activity and function. Based on the advancing technologies of sequencing and analyses, genes related to secondary metabolites biosynthesis, transport, and catabolism of *G. leucocontextum* were obtained, and the number of some specific genes in *G. leucocontextum* was much more than other edible and medical fungus; the relationship between these genes and the biological characteristics and pharmacological activities remains to be further studied. Like the rich gene of CEs in *G. leucocontextum*, we speculate that this characteristic was to better adapt to the special climate of the plateau. As a newly identified member of *Ganoderma*, there will be abundant of active ingredients and metabolic genes to be excavated and utilized. Although incomplete, the results of *G. leucocontextum* genome in this study provide a preliminary insight to the biosynthesis of active secondary metabolites and can be used as a theoretical reference for the development and application of *Ganoderma* industry.

## Author statement

The authors declare that the research was conducted in the absence of any commercial or financial relationships that could be construed as a potential conflict of interest. All the authors have seen the manuscript and approved to submit to your journal. Neither the entire paper nor any part of its content has been published or has been accepted elsewhere. It is not being submitted to any other journal as well.

## Data availability

The assembled genome sequence of *G. leucocontextum* has been provided to NCBI with the BioProject ID PRJNA729903 and accession number JAHKGY000000000. Supplementary material is available at figshare: https://doi.org/10.25387/g3.16545636.
